# Meals with Less Meat

**Published:** 1912-10-19

**Authors:** 


### INSTITUTIONAL HOUSEKEEPING.

### Meals with Less Meat.

It is common to imagine that a meatless diet means subsisting solely on vegetables, and, after considering the many and just reasons against such a diet, it is concluded there is no alternative to the customary joints, soups, etc. Yet it is recognised by an increasing number of thinking people that the amount of meat consumed by the upper and middle classes in this country is in excess of what is required for the attainment of physical and mental vigour. As meat, too, is an expensive and wasteful food, the economic side of meat-eating is attracting more and more attention. The main difficulty in reforming this extravagant national habit lies in providing a satisfactory substitute. A mass of badly cooked vegetables, usually with the juices and flavour carefully extracted by boiling, is worthless for body-building and repairing. Since quality not quantity is required for adequate nutrition, the common complaint made against vegetarianism—that it involves the consumption of an extra large bulk of food—would constitute a very serious objection were it true. It would be ridiculous to advocate a non-meat diet for institutions, including as they do persons of very varied tastes and all engaged in strenuous labours, but it is well worth considering what variety could be gained and what money saved by a modification of the meat-eating habits, handed down to us by custom as descendants of meat-eaters.

The systematic introduction at each meal of one or two sustaining and appetising dishes in which no form of animal food entered would certainly prove popular, for matrons constantly comment on the welcome given by members of their staff to such preparations as cauliflower *au gratin*, nut cutlets, macaroni cheese, spinach and eggs, and other homely dishes. Tired workers, especially those engaged in nursing the sick, often experience a revulsion against roast or boiled meat, though compelled to eat it for lack of something more attractive; yet the caterer considers her duty done and all cause for grumbling removed if meat be provided in sufficient quantities. Supposing one meatless item only was inserted in the menu instead of the usual choice of joint and made-up dish. It might consist of eggs in some simple form combined with vegetables or cheese; or, as eggs do not agree with all, the main ingredient might be cheese with macaroni, spaghetti, or rice. It must be remembered that cheese grated and cooked is infinitely superior, from the digestive point of view, to a lump of cheese uncooked. To facilitate its common use a cheese and nut mill or grater will be found most useful wherever the enterprising matron intends full advantage to be taken of these valuable foods. Pulse foods, really well cooked and served curried, stewed, or with sauce, are most valuable, brown haricots being particularly savoury and too little known and used.

A point often brought forward against lessening the quantity of meat in the diet is that the proteids derived from meat are more easily digested by many persons than vegetable proteids. But against this must be reckoned the fact that the latter proteids and those derived from milk and milk products do not undergo the same process of putrefaction which for weak digestions is apt to render a meat diet harmful. That a large amount of meat is necessary to sustain mental and physical vigour is disproved by an examination of the national foods of many races renowned for their powers of endurance, activity, and longevity. Arabs live largely on milk and dates; Greek fishermen on black bread, figs, and grapes or raisins; Norwegians on rye bread, milk, and cheese. And how much meat is procurable by our own agricultural labourers on 15s. a week? Matrons and others in charge of the catering department must not imagine that by providing a substitute for meat the arrangement of menus will be simplified. At the beginning of reform caterers commonly make mistakes through ignorance or carelessness in balancing their menus, and thus provide on one day a surfeit of proteid foods, on the next nothing but comparatively unnutritive dishes. Another important point often overlooked is that non-meat dishes require to be served even more thoroughly hot than those compounded of meat or fish, for eggs, cheese, and dishes cooked with butter cool quite as speedily and unpleasantly as the fat of meat. New eating habits are acquired slowly, but if for a few weeks just one simple meatless dish is introduced at each meal and its cost and the reception it meets with be carefully noted, the innovation will probably become permanent, and will contribute to the health of the inmates and the reduction of the cost of living.

### Housekeeping Queries.

X. Y. Z.—To prevent macaroni sticking to the pan whilst boiling find a wire sieve that will slip easily into a large saucepan, put the macaroni into this, and then slip it down into the boiling water. When the macaroni is cooked the sieve can easily be raised and the water be perfectly drained off as well as all risk of burning being prevented.

Damp Walls.

Sister Clare.—The reason why the walls of your corridors and staircases are "running with water" is the sudden changes in the weather. Lately the nights have been very chilly, and corridors become cold, then when the outside air becomes warm and oppressive everyone throws open doors and windows. The warmer the air the greater the amount of vapour present, and when this comes in contact with the cold walls condensation takes place and the liquid is deposited over the surface. The same thing happens if a jug of really cold water is placed in a warm room. The jug will be covered with dew, not because it is porous, but because the chilled surface condenses the watery vapour in the room. It must, however, be remembered that the precipitation of moisture on the walls, etc., has the same effect as if it oozed through them, for it loosens and discolours papers, makes the beds damp, and renders fabrics limp and musty. Hence it is well to avoid the sudden opening of windows and doors when the atmosphere outside is full of moisture and warmer than the interior of the house.

